# Carbon emissions and sustainability in Covid-19’s waves: evidence from a two-state dynamic Markov-switching regression (MSR) model

**DOI:** 10.1007/s10479-023-05184-x

**Published:** 2023-02-04

**Authors:** Konstantinos N. Konstantakis, Panayotis G. Michaelides, Panos Xidonas, Stavroula Yfanti

**Affiliations:** 1 The Laboratory of Theoretical and Applied Economics and Law, The School of Applied Mathematical and Physical Sciences, National Technical University of Athens, Athens, Greece; 2 Hellenic Open University, Patras, Greece; 3 ESSCA Ecole de Management, Paris, France; 4 University of London, Queen Mary, London, UK

**Keywords:** Environment, Sustainability, Pandemic, CO_2_, C22, C58, C50, C51

## Abstract

Throughout the world, carbon emissions have decreased in an unprecedented way as a result of the Covid-19 pandemic. The purpose of this paper is to investigate whether a rebound effect in carbon emissions is anticipated following the extraction of information related to the beliefs of investors. A suitable Markov switching model is used in this paper to adapt the safe haven financial methodology to an environmental sustainability perspective. Analytically, the aforementioned situation is modeled by estimating a two-state dynamic Markov-Switching Regression (MSR), with a state-dependent intercept term to capture the dynamics of the series, across unobserved regimes. In light of the results of the research and the robustness checks, investors are anticipating a rebound effect on the total quantity of carbon emissions.

## Introduction

According to Zhao et al. (2020), climate change and environmental pollution have attracted increasing attention lately in the research agenda of various authors (see, among others, Martin et al., 2014, and Han et al., 2019). In this context, the carbon emission trading (CET) market constitutes a financial market, which aims at reducing carbon emissions and controlling climate change that has also been a hot research topic lately for both economics and operations academic literature (Allevi et al., 2017; Boutabba & Lardic, 2017; Du et al., 2020; Fang & Ma, 2021; Oestreich & Tsiakas, 2015; Song et al., 2015; Tang et al., 2017).

Meanwhile, the financial community still struggles to understand and evaluate the magnitude of the damages caused by the recent Covid -19 pandemic, at a time when several major assets have lost part of their initial value. However, since the beginning of the Covid-19 pandemic spread, carbon emissions values have risen. This is quite impressive given the losses suffered by other assets in the first wave of the pandemic. In the light of this new era, scientists across various disciplines try to cope with the unexpected phenomena induced by the pandemic itself. As a result, researchers in the field try to analyse and assess the impact of the pandemic on hazardous emissions and especially on emissions that contribute essentially to the Green House Effect (GHE). Based on official statistics, in 2020 the CO_2_ emissions experienced a reduction equal to 7% compared to 2019, the largest in the post industrial era (Friedlingstein et al., 2020). This reduction in CO_2_ emissions is attributed to the reduction of the overall economic activity due to the unprecedent lockdown measures implemented by the majority of economies across the globe, induced as a last resort measure for the containment of the Covid-19 virus, mainly for the protection of public health.

Despite the fact that most economies faced a tremendous recessionary impact because of the pandemic, they withnessed an overwhelming reduction in their daily CO_2_ emissions that exceeded 17% compared to 2019, and peaked at almost 23% reduction, when the confinement measures were in their peak (Quere et al., 2020). In fact, the total dropdown in carbon emissions for 2020 was estimated to be approximately equal to 6.7% (Tolleson, 2021). In this context, a question of paramount importance is whether this reduction in CO_2_ emissions is expected to be sustained and which policy actions would be appropriate to eliminate a potential rebound effect of the carbon dioxide emissions in the post-pandemic era.

In order to sufficiently tackle this research question, we need to extract information regrading the expectations of the future levels of Carbon Dioxide emmisions. To do so, in this paper we will make use of the future returns of CO_2_ emissions that are freely traded in the financial markets. In fact, in this work we will examine whether in the pandemic era the CO_2_ futures acted as a safe-haven alternative to either the stock market index or the 10-year US bonds yields, discriminating the preferences of investors across the two waves of the Covid-19 pandemic. Based on our findings, we will indirectly extract the information needed regrading the future level of CO_2_ emissions in the post- pandemic era.

The exctraction mechanism is as follows: *If the CO*_*2*_* futures are found to act as a safe-haven then investors expect that in the future their price will rise. This, in turn, implies that the demand for futures of CO*_*2*_* emissions will rise. This rise in demand will be based on two demand components, the first component is the rise due to speculation whereas the second component is the rise due to the increased demand of non-efficient firms that need to obtain an increased share of “polluting” licences in order to maintain their level of CO*_*2*_* emissions, without having to invest into more efficient environmentally friendly technologies of production. Of course, from the supply side we have to acknowledge the fact that due to the lockdown measures, consumption of economies has hindered and as a result production has decreased. This, in turn, offered the insentive to efficient non-polluting firms to increase the supply for futures of CO*_*2*_* emissions, whereas non-efficient firms increased their repective demand for these futures in order to delay their investments into environmentaly healthy technologies.*

According to Tan et al. (2020), owing to the weak interactions between the carbon market and other conventional markets, carbon assets provide diversification and hedge benefits, especially during periods of market turmoil (Koch, 2014). However, thus far, we observe a notable gap in the extant literature with a dearth of studies explicitly examining the role of carbon emissions, in the two recent covid-19 waves. In addition, we look at investor reactions to the varying intensity of the current pandemic. Our dataset allows us to differentiate between the pandemic effects of various sizes, in terms of volatility. The investigation covers a 12-month period, from 1 January 2020 to 1 January 2021, using daily data.

In this work, we use relevant Markov switching techniques in order to investigate the aforementioned questions in a high and low volatility state, respectively. More precisely, we allow the data to be characterized by two states, namely a high-mean state, which represents the market expectation of more volatile returns, and a low-mean state, which represents low volatility expectations (Burdekin and Tao, 2021). The aforementioned situation is modeled by estimating a two-state dynamic Markov-Switching Regression (MSR), with a state-dependent intercept term to capture the dynamics of the series, across unobserved regimes.

In brief, our paper advances the literature in the following ways: (a) It is the first study that adapts the safe-heaven hypothesis to the specific research question, to the best of our knowledge; (b) it uses state-of-the-art Markov Switching (MS) techniques to empirically assess the aforementioned behaviour; (c) it comparatively examines the recent pandemic’s two waves on carbon emissions as a safe haven, extracting substantial information for the expectations of investors; (d) it produces policy implications for practioners that could be directly used for the implementation of tailor-made actions that will ensure the permanent reduction of carbon emissions.

The paper is structured as follows: Sect. 2. offers a review of the recent literature on carbon emissions as a safe haven; Sect. 3 sets out the methodological framework; Sect. 4 contains the empirical analysis; Sect. 5 discusses the results; finally, Sect. 6 concludes the paper.

## Literature review

The literature review covers two distinct and relevant strands The first strand analyses the related empirical literature of carbon emission markets, whereas the second strand covers the empirical literature on safe haven assets.

### Carbon emissions markets

Most theorists and empiricists explore the properties of emissions as a new commodity or financial asset (given the commodity financialization hypothesis) and delve into the relationship of this novel asset class with other more traditional investment areas (see, for example, Hammoudeh et al., 2014) either commodities (e.g., energy, metal) or pure financial instruments (e.g., stocks, bonds). A further strand of the literature investigates the macro-relevance of emissions by connecting their price pattern to economic fundamentals or overall market conditions (for instance, comparing crisis versus tranquil periods of financial markets).

Among the early studies trying to investigate the stylized facts of emissions trading, Oberndorfer (2009) shows that European Union Allowances (EUAs) price changes and stock returns of several important European corporations are positively related. Chevallier (2009) demonstrates that carbon futures returns are mostly associated with power demand and allowances supply and only weakly related to macroeconomic fundamentals in contrast to the large bulk of commodities. However, in a later study, Chevallier (2012) provide strong empirical evidence of time-varying pairwise correlations between carbon prices, oil, and gas.

In a further attempt to connect emissions with conventional financial assets, Kumar et al. (2012) prove a weak relationship between carbon and stock prices of clean energy firms. Moreover, Reboredo (2013) examines the dependence structure between EUAs and crude oil markets, during the second commitment period of the European Union Emissions Trading Scheme (EU ETS) and finds that the EUA market is an attractive market for investors in terms of diversifying market risk and reducing the downside risk of crude oil markets. In this vein, Koch (2014) explores the linkages among carbon, energy, and financial markets and reveals a much closer carbon-energy price linkage in the second phase of the EU ETS. Similarly, Sousa et al. (2014) analyze the interrelation of carbon prices with energy prices and economic activity and find that these relations are becoming stronger, and then disappear over distinct time intervals and frequencies. Furthermore, Boersen and Scholtens (2014) show that energy assets are significant drivers of the carbon futures price. Turning to the second moment of emissions time series pattern, Marimoutou and Soury (2015) examine the volatility dependence structure between carbon dioxide emissions and energy prices. They prove that their dependence varies over time, remaining rather stable in tranquil periods but significantly rising during crises.

Oestreich and Tsiakas (2015) further scrutinize the role of the European emissions trading system on German stock returns. They witness that firms, which received free carbon emission allowances, significantly outperformed firms that did not. Zheng et al. (2015) uncover a significant cross-correlation between stock markets, energy, and financial futures. Hammoudeh et al. (2015), using a Nonlinear Autoregressive Distributed Lag (NARDL) model, analyze the effects of energy assets on emission allowance prices and estimate a long-run negative asymmetric impact. Tian et al. (2016) argue that the relationship between the EUA market and stock returns of electricity companies is largely driven by strong market shocks. Moreover, the stock volatility of electricity companies is significantly driven by EUA market fluctuations in the same direction, whereas stock returns of carbon-intensive companies are negatively affected by the EUA returns. Wei and Lin (2016) investigate the link between carbon, oil, and stock index futures. Their results indicate that carbon futures returns respond to oil shocks, whereas the oil market has an impact on the volatility of the other two markets, but it is much less affected by them.

More recently, Wen et al. (2017), discuss that despite the superiority of hedged portfolios in increasing the risk-adjusted returns of carbon assets, the dynamic diversified portfolios are much preferred for reducing variance and the downside risks of carbon assets. Cong and Lo (2017) show that the rate of return in the Chinese emissions market is negatively associated with expected risk. According to Jiang et al. (2018), coal, oil, and stocks have a negative impact on the carbon price, while in the special case of European markets there is strong causality running from European stocks to the EUA prices (Jiménez-Rodríguez, 2019).

In brief, our literature review is consistent with the seminal work by Tan et al. (2020), who are the first to empirically formalize the “Carbon-Energy-Finance” system by connecting the carbon market with commodity, stock, and bond markets via (a) the correlated-information channel (i.e. “return spillover”), through which connections occur based on prices (Kodres & Pritsker, 2002); and (b) the risk premium channel (i.e. “volatility spillover”), through which a shock in one market may adversely affect any other market (Acharya & Pedersen, 2005).

In conclusion, the carbon emission allowances are tightly linked to other energy and non-energy assets and have been fast becoming an investment area, with a relatively mature and continuously growing market that is attractive to investors in terms of diversifying and mitigating risk.

### Safe haven assets

The safe-haven hypothesis is introduced in the relevant literature by Baur and Lucey (2010) in an attempt to investigate whether gold acts as safe haven in periods of crisis and increased volatility. They study constant and time-varying relations between U.S., U.K. and German stock and bond returns and gold returns and find that gold is a hedge against stocks on average and a safe haven in extreme stock market conditions. Joy (2011), using a model of dynamic conditional correlations covering 23-years of weekly data for 16 major dollar-paired exchange rates shows that, during the past 23-years, gold has behaved as a hedge against the US dollar and as a poor safe haven.

Hood and Malik (2013) evaluate the role of gold relative to volatility (Volatility Index (VIX)) as a hedge and safe haven. Using daily data from the US stock market, it is shown that gold serves as a hedge and a weak safe haven for US stock market. However, it seems that in periods of extremely low or high volatility, gold does not have a negative correlation with the US stock market.

Bredin et al. (2015), utilising wavelet analysis, find that gold acts as a hedge for a variety of international equity and debt markets for horizons of up to one year and that gold acts as a safe haven for equity investors for long-run horizons of up to one year. However, during the economic contractions of the early 1980s, gold displayed a positive relationship with equities across a range of horizons.

Beckmann et al. (2015), test the Baur and Lucey (2010) hypothesis, by augmenting their model to a smooth transition regression (STR) using an exponential transition function which splits the regression model into two extreme regimes, and including in their study a set of 18 individual markets as well as 5 regional indices between 1970 and 2012 monthly. Their findings show that gold serves as both a hedge and a safe haven.

Baur and McDermott (2010a, b) show that gold is a particularly strong safe haven in the aftermath of September 11, 2001 and the Lehman bankruptcy in September 2008. Chkili (2016) examines the dynamic relationships between gold and stock markets, using data for the BRICS counties, and shows that, during the major financial crises, gold can act as a safe haven against extreme market movements. The same author, Chkili (2017), uses the Markov switching approach to show that gold can act as a weak hedge and a strong safe haven against extreme Islamic stock market movements.

Chen and Wang (2017), examine the dynamic relationships between gold and stock markets in China. Using daily gold and stock indexes data, showed that gold acted as a safe haven for only the latest two of the five bear markets analyzed, whereas for non-bear markets, gold does not offer good risk hedging. Wen and Cheng (2018) find that while both gold and the US dollar can serve as a safe haven for emerging stocks, the latter is better than gold in most cases and that its superiority in hedging infinitely extreme risks is weakened in the subsample of the global financial crisis.

Chen and Wang (2017) aim to examine the hedge and safe haven properties of gold relative to Dow Jones stock industry indices. Their results show that the hedge and safe haven properties of gold have a changing nature. During 1980–2017, gold is a safe haven for almost all sectors, while during the sub-periods, the properties of Gold as a hedge and a safe haven vary.

Ji et al., (2020) in their paper attempt to re-evaluate the safe-haven role of some traditional asset types, namely, gold, cryptocurrency, foreign exchange and commodities and their results show that gold commodity futures remain robust as safe-haven assets during this pandemic.

Boubaker et al. (2020), using annual data spanning the period 1258–2018, test the safe haven characteristic of gold in the wake of global crises. It is argued that, under certain conditions, gold serves as a strong hedge against crises, especially during the bullish regime of the market, and in particular from the post-World War I period, while global crises can accurately predict real gold returns over a long-span (1302–2018) out-of-sample period.

Dutta et al. (2020) investigate the time-varying correlations between gold and oil markets to examine whether gold is a safe haven asset for the international crude oil markets during the Covid-19 period. According to their results gold is a safe haven asset for global crude oil markets. Gharib et al. (2020) examine the causal relationship between crude oil and gold spot prices to assess how the economic impact of Covid-19 has affected them. They detect common periods of mild explosivity in WTI and gold markets and also find a bilateral contagion effect of bubbles in oil and gold markets during the recent Covid-19 outbreak.

As recently as 2022, there has been a study by Madani and Ftiti (2022) which investigated whether gold could serve as a hedge against oil price fluctuations or currency movement regardless of calm or extreme market conditions. As part of the empirical analysis, thet extend the intraday multifractal correlation measure developed by Madani et al. (Bankers, Markets & Investors, 163:2–13, 2020) so as to take into account the dependence of calm and extreme price movements across different time frames. To examine the time-varying relationship between gold-oil and gold-currency under calm and turbulent market conditions, they use the rolling window method. The analysis of high frequency (5-min intervals) data over the period 2017–2019 reveals three interesting findings. Firstly, gold acts as a weak (strong) hedge against oil (currency) market movements. Second, gold has strong safe-haven capabilities against extreme currency fluctuations and against only short-term fluctuations in oil prices. Third, hedging strategies confirm that gold is an effective hedge or safe haven for portfolio risk reduction. Finally, the paper discusses the implications for investors, financial institutions, and policy makers.

Furthermore, several studies have examined the role of gold as a hedge or safe-haven asset and recently Huynh et al. (2020a, b) examined the informational linkage between cryptocurrency markets and gold (and oil). To hedge against unexpected movements in the cryptocurrency (oil) market, investors should rebalance their portfolios by including gold (cryptocurrency). Furthermore, Thampanya et al. (2020) investigated the hedging effectiveness of gold and bitcoin for equities using the linear and non-linear Autoregressive Distributed Lag (ARDL) framework. According to their research, most of the effects of gold on the stock market can be characterized as asymmetric.

In brief, the literature on safe haven assets is primarily focused on the role of gold, with very few exceptions. As a result, the present paper is the first to the best of our knowledge that utilizes the safe haven methodology for Carbon emmisions.

## Methodology

In what follows, we will briefly set out the methodology to test the safe-haven hypothesis, regarding carbon emissions.

### Hypothesis formulation

Based on the seminal work of Baur and Lucey (2010), we begin by defining three different states of an asset in an investment portfolio (see, also, Mensi et al., 2016, Balcilar et al., 2016, and Selmi et al., 2018).

#### ***Definition 1 (Hedge)***

*An asset that is uncorrelated or negatively correlated with another asset is defined to exhibit a hedge behavior.*

#### ***Implications of Definition ******1***

In an environmental sustainability perspective, if carbon emmisions exhibit a hedge behaviour, then investors expect that in the future the price of emissions will rise. This increase is attributed to the increase in demand for the asset due to speculation and due to the expected increase of carbon emissions. The expected increase in the carbon emmisions could be attributed to firms that either delayed their investments to eviromentally friendly technologies (due to their inaction during the pandemic) or to firms that intentionally try to exploit the low price of carbon emmisions now in order to use “polluting” licences in the future. Irrespectively of the case, the information drawn is that the expected price of carbon emmisions will rise in the future, a fact that in turn implies that the expected total quantity of carbon emmisions will also increase in the future.

##### ***Definition 2 (Diversifier)***

*An asset that is positively but not perfectly correlated with another asset is defined to exhibit a diversifier behavior.*

#### ***Implications of Definition ******2***

In an environmental sustainability perspective, if carbon emmisions exhibit a diversifier behaviour, then we cannot have a valid inference regrading the expectations of investors. Therefore, in this case, no indirect inference regrading the future price of carbon emmisions is drawn, which, in turns, implies that no inference regarding the expected total quantity of carbon emmisions is drawn.

##### ***Definition 3 (Safe haven)***

*An asset that is uncorrelated or negatively correlated with another asset in times of extreme financial turmoil is defined to exhibit a safe-haven behaviour.*

#### ***Implications of Definition ******3***

According to the extant financial empirical literature, a safe haven is considered as an asset that does not lose its initial value in times of crises or during bearish market conditions and helps investors in protecting their wealth in turbulent times. A strong safe-haven asset is negatively related to the reference asset or portfolio and therefore gains value as the reference asset loses value (Baur & McDermott, 2010a, b). In an environmental sustainability perspective, if carbon emissions exhibit a safe heaven behavior, then investors expect that in the future the price of emmissions will rise in contrast to other assets or commodities. This expected increase in the future price of carbon emissions is translated as an expected future increase in the quantity of the carbon emissions.

It is worth noticing that the implications derived by definition 1 and 3 are quite similar. The sole difference lies in the fact that the expectations derived by definition 3 are stronger than those of definition 1. Nonetheless, from an environmental sustainability percepective, we are only interested to extract information of the future beliefs (expectations) of investors that will lead us to infer expectations regarding the future total quantity of the carbon emissions. In this context, the implications regarding the future expectations of quantity of future emissions are practically the same across the two definitions.

### Model building: markov switching

Following Baur and Lucey (2010), we define the equation that will be used in order to test the safe-haven property of our asset as:

1 $$ Y_{t} = a_{0} + \Phi \left( {L_{1} } \right)Y_{{t - L_{1} }} + AX_{t} + \Phi \left( {L_{2} } \right)X_{{t - L_{2} }} + B{\rm X}_{t. q\left( a \right)} + \varepsilon_{t} $$

where: ${Y}_{t}$ is the asset under investigation that we wish to uncover its behaviour according to the definitions provided earlier, $\Phi \left(L\right)$ is a vector of lag coefficients of the asset, ${X}_{t}$ is a vector of competing assets against which the behaviour of the ${Y}_{t}$ asset is examined, A is a vector of the respective coefficients,$\Phi \left({L}_{2}\right)$ is the vector of the lagged coefficient of the competing assets, ${\rm X}_{t. q\left(a\right)}$ is a vector that accounts for asymmetries of positive and negative extreme shocks in the competing assets of a% lower quantile q, thus it takes the value of zero if the returns of the competing asset(s) are larger than the a% quantile, and the value of one (1) elsewhere, B is the vector of the respective coefficients.

In order to account for the two different regimes (asymmetries) in the volatility of an asset, we make use Markov-Switching (MS) regimes. Therefore, by making the assumption that all the variables in our model are state-dependent, the Eq. (1) is transformed to a Markov Switching Regime equation as follows:

2 $$ Y_{t} = \alpha_{{S_{t} }} + \Phi_{{S_{t} }} \left( {L_{1} } \right)Y_{{t - L_{1} }} + A_{{S_{t} }} X_{t} + \Phi_{{S_{t} }} \left( {L_{2} } \right)X_{{t - L_{2} }} + B_{{S_{t} }} {\rm X}_{t. q\left( a \right)} + \varepsilon_{t} $$

where ${s}_{t}$ is a random variable that result in changes happening in the sample to assume the value ${s}_{t}=1$ for $t={t}_{0}+1,{t}_{0}+2, \dots$ The description of the probability law governing the observed data would require a probabilistic model explaining the change from ${s}_{t}=1$ to ${s}_{t}=2$. The simplest specification is the realization of a two-state Markov chain with:

3 $$ \Pr \left( {s_{t} = j{|}s_{t - 1} = i, s_{t - 2} = k, \ldots .,y_{t - 1} ,y_{t - 2} , \ldots } \right) = \Pr \left( {s_{t} = j{|}s_{t - 1} = i} \right) = p_{ij} $$

Therefore, for the two different regimes we have the following regression equation:

4 $$ Y_{t} = \left\{ {\begin{array}{*{20}c} {\alpha_{1} + \Phi_{1} \left( {L_{1} } \right)Y_{{t - L_{1} }} + A_{1} X_{t} + \Phi_{1} \left( {L_{2} } \right)X_{{t - L_{2} }} + B_{1} {\rm X}_{t. q\left( a \right)} + \varepsilon_{t,1} , \varepsilon_{t,1} \sim N\left( {0,\sigma_{1}^{2} } \right) if\, s_{t} = 1} \\ {\alpha_{2} + \Phi_{2} \left( {L_{1} } \right)Y_{{t - L_{1} }} + A_{2} X_{t} + \Phi_{2} \left( {L_{2} } \right)X_{{t - L_{2} }} + B_{2} {\rm X}_{t. q\left( a \right)} + \varepsilon_{t,2} , , \varepsilon_{t,2} \sim N\left( {0,\sigma_{2}^{2} } \right) if\, s_{t} = 2} \\ \end{array} } \right. $$

The parameters necessary to describe the probability law governing ${y}_{t}$ are the variances of the Gaussian innovation ${\sigma }_{1}^{2}$ and${\sigma }_{2}^{2}$, the vectors of autoregressive coefficients ${\Phi }_{1}\left({L}_{1}\right)$ and${\Phi }_{2}\left({L}_{1}\right)$, the two intercepts ${\alpha }_{1}$ and${\alpha }_{2}$, the coefficient vectors of the control variables ${A}_{1}$ and${A}_{2}$, the respective lagged coefficient vectors of the control variables ${\Phi }_{1}\left({L}_{2}\right)$ and ${\Phi }_{2}\left({L}_{2}\right)$, the coefficient vectors of the quantile control variables ${B}_{1}$ and ${B}_{2}$ and the two state transition probabilities ${p}_{11}$ and${p}_{22}$.

Note that the probability of a change in regime depends on the past only through the value of the most recent regime (Hamilton, 1994). Suppose that ${Y}_{t}$ is observed directly and the value of ${s}_{t}$ is based on what we see happening with ${y}_{t}$. Then we have the probabilities:

5 $$ \xi_{it} = {\text{Pr}}(s_{t} = j|\Omega_{t} ;\theta ) $$

For j = 1,2 where these two probabilities sum to unity. ${\Omega }_{t}=\{{y}_{t},{y}_{t-1},\dots ,{y}_{1},{y}_{0}\}$ and denotes the set of observations obtained as of date $t$, $\theta$ is a block vector of population parameters:

i.e.. $$\theta = \left( {\alpha_{1} ,\alpha_{2} , \Phi_{1} \left( {L_{1} } \right), \Phi_{2} \left( {L_{1} } \right), A_{1} , A_{2} , \Phi_{1} \left( {L_{2} } \right), \Phi_{2} \left( {L_{2} } \right), B_{1} , B_{2} ,p_{11} ,p_{22} } \right)^{^{\prime}}$$

The inference is performed iteratively for t = 1,2,…,T, tilth step t accepting as input the values:

6 $$ \xi_{i,t - 1} = {\text{Pr}}(s_{t - 1} = i|\Omega_{t - 1} ;\theta ) $$

For i = 1,2. The key magnitudes needed in order to perform this iteration are the densities under the two regimes:

7 $$  \eta_{it} = \Pr \left( {y_{t} {|}s_{t}  = j,\Omega_{t - 1} ;\theta } \right) = \frac{1}{{\sqrt {2\pi } \sigma }}\exp \left\{ { - \frac{{\left( {y_{t} - \alpha_{{S_{t} }} - \Phi_{{S_{t} }} \left( {L_{1} } \right)Y_{{t - L_{1} }} - A_{{S_{t} }} X_{t} - \Phi_{{S_{t} }} \left( {L_{2} } \right)X_{{t - L_{2} }} - B_{{S_{t} }} {\rm X}_{t. q\left( a \right)} } \right)^{2} }}{{2\sigma^{2} }}} \right\} $$

For j = 1,2. We then can calculate the conditional density of the t-th observation from the following equation:

8 $$ f\left( {y_{t} {|}\Omega_{t - 1} ;\theta } \right) = \mathop \sum \limits_{i = 1}^{2} \mathop \sum \limits_{j = 1}^{2} \eta_{jt} p_{ij} \xi_{i,t} $$

Then, we derive:

9 $$ \xi_{i,j} = \frac{{\mathop \sum \nolimits_{i = 1}^{2} \eta_{jt} p_{ij} \xi_{i,t - 1} }}{{f\left( {y_{t} {|}\Omega_{t - 1} ;\theta } \right)}} $$

As a result of executing this iteration, we may succeed in evaluating the sample conditional log likelihood of the observed data:

10 $$ {\text{log}}f\left( {y_{1} ,y_{2} , \ldots ,y_{T} {|}y_{0} ;\theta } \right) = \mathop \sum \limits_{t = 1}^{T} {\text{log}}f\left( {y_{t} {|}\Omega_{t - 1} ;\theta } \right) $$

For the specified value of θ, an estimate of the value of θ can then be obtained by maximizing (10) by numerical optimization. For the value ${\xi }_{i0}$ to use to start these iterations. If the Markov chain is presumed to be ergodic, we can use the unconditional probabilities:

$${\xi }_{i0}=\mathrm{Pr}\left({s}_{0}=i\right)=\frac{1-{p}_{jj}}{2-{p}_{ii}-{p}_{jj}}$$

Let ${\Omega }_{t}=\{{y}_{t},{y}_{t-1},\dots , {y}_{1}\}$ be the observations through date t, P be a (N x N) matrix whose row j, column I is the transition probability ${p}_{jj}$, ${\eta }_{t}$ a (N × 1) vector whose jth element

11a $$ f\left( {y_{t} {|}\Omega_{t - 1} ;\theta } \right) = 1^{\prime}\left( {P\widehat{{\xi_{t - t|t - 1} }} \odot \eta_{t} } \right) $$

11b $$ \widehat{{\xi_{t|t} }} = \frac{{P\widehat{{\xi_{t - t|t - 1} \odot \eta_{t} }}}}{{f\left( {y_{t} {|}\Omega_{t - 1} ;\theta } \right)}} $$

where 1 denotes an (N × 1) vector all of whose elements are unity and $\odot$ denotes element by element multiplication.

A specification where the density depends on a finite number of previous regimes, $f\left({y}_{t}|{s}_{t},{s}_{t-1},\dots ,{s}_{t-m},{\Omega }_{t-1};\theta \right)$ can be recast in above form, by a suitable definition of regime (Hamilton, 1994). In the empirical analysis, we apply the aforementioned methodology and derive the Maximum Likelihhod estimates empirically.

### Dating of pandemic waves

#### Dating using BSADF

The method is introduced in the literature by Phillips et al. (2011) (PWY) and was extended by Phillips et al. (2015) (PSY). However, since then, the method has been further developed by Michaelides, Tsionas and Konstantakis (2016), Phillips και Shi (2020). The method builds on the modified unit root test of Dickey και Fuller (1979), and is based on the following equation:

12 $$ \Delta y_{t} = a_{{r_{1,} r_{2} }} + b_{{r_{1,} r_{2} }} y_{t - 1} + \mathop \sum \limits_{i = 1}^{K} \delta_{{r_{1,} r_{2} }}^{i} \Delta y_{t - i} + \varepsilon_{t} $$

where $\Delta$ is the first difference operator, ${y}_{t}$ is the time series variable that exhibits explosive behavior, $t$ is the time dimension, ${\rm K}$ denotes the number of and ${r}_{1,}{r}_{2}$ denote the beginning and the end of the estimation period, repsecively. In this set up, in case there are ${\rm T}$ time periods in the sample then ${r}_{1}$ and ${r}_{2}$ could be expressed as parts of ${\rm T}$ such that:

13 $$ r_{2} = r_{1} + r_{w} $$

where ${r}_{w}$ is the estimation window. Therefore, the sample size for the estimation of Eq. (12) is:

14 $$ {\rm T}_{w} = \left\lfloor {T_{{r_{w} }} } \right\rfloor $$

where $\left\lfloor . \right\rfloor$ is the integer function. The hypothesis tested using the methodology described is:

$$\left(\genfrac{}{}{0pt}{}{{\rm H}_{0}: {b}_{{r}_{1,}{r}_{2}}=0 (\mathrm{unit\, root\, existence})}{{\rm H}_{1}: {b}_{{r}_{1,}{r}_{2}}>0 (\mathrm{explosive\, behavior})}\right)$$

For simplicity let the t-statistic used for the null hypothesis (${\rm H}_{0})$ testing be the ${ADF}_{{r}_{1}}^{{r}_{2}}$. In this context, based on Phillips et al. (2011), two statistics need to be estimated. The first statistic is ADF right-tailed statistic which is based on the number of observations such that ${r}_{1}=0$ and ${r}_{2}=1$ which in turn yields that ${r}_{w}=1$, is denoted with ${ADF}_{0}^{1}$.The second statistic, which is called Supremum ADF (SADF), is based on the supremum of the t-statistic of a forward recusive estimation of Eq. (12) of the form:

15 $$ SADF\left( {r_{0} } \right) = sup_{{r_{2} \in \left[ {r_{0} ,1} \right]}} \left\{ {ADF_{0}^{{r_{2} }} } \right\} $$

Finally, in case of multiple bublles in the estimation sample, PSY introduced the Backward Supremum ADF statistic of the form:

16 $$ BSADF_{{r_{2} }} \left( {r_{0} } \right) = sup_{{r_{1} \in \left[ {0,r_{2} - r_{0} } \right]}} \left\{ {ADF_{{r_{1} }}^{{r_{2} }} } \right\} $$

For the dating purposes of the multiple Covid-19 waves we will base our analysis on BSADF.

#### Dating using structural break test

In this work we make use of the Bai and Perron (1998) structural break test which was extended by Bai and Perron (2003) and Ditzen (2018). The test for $T$ periods and $S$ structural breaks is based on the following equation:

17 $$ y_{t} = bx_{t} + \delta_{j} w_{t} + \varepsilon_{t} $$

where $t={T}_{j-1},\dots ,{T}_{j}$ and $j=1,\dots ,s+1$ with ${T}_{0}=0$ and ${T}_{s+1}=T$. Hence there are $s$ breaks, or $s+1$ regimes with regime $j$ covering the observations ${T}_{j-1},\dots ,{T}_{j}$. In this set up, the vector of regressors ${x}_{t}$ are unaffected by the structural breaks whereas the ${w}_{t}$ regressors are affected by the breaks.

In order to test for a specific number of structural breaks in our sample we make use of the following hypothesis set:

$$\left(\begin{array}{c}{H}_{0}:s breaks\\ {H}_{1}:s+1 breaks\end{array}\right)$$

If we assume that the set of structural break dates is ${T}_{s}=\{{\widehat{T}}_{1},\dots ,{\widehat{T}}_{s}\}$ then the statistic used for testing the null hypothesis is:

18 $$ F\left( {s + {\raise0.7ex\hbox{$1$} \!\mathord{\left/ {\vphantom {1 s}}\right.\kern-0pt} \!\lower0.7ex\hbox{$s$}}} \right) = sup_{1 \le j \le s + 1} sup_{{\tau \in \hat{T}_{j,\varepsilon } }} F\left( {\tau \backslash \hat{T}_{s} } \right) $$

where $\widehat{{T}_{s}}$ contains estimates of the s break stipulates under the null hypothesis, $\tau$ is the additional $(s+1)$-th break under the alternative, and

$$\widehat{{\rm T}_{j,\varepsilon }}=\{\tau :\widehat{{\rm T}_{j-1}}+\left(\widehat{{\rm T}_{j}}-\widehat{{\rm T}_{j-1}}\right)\varepsilon \le \tau \le \widehat{{\rm T}_{j}}-\left(\widehat{{\rm T}_{j}}-\widehat{{\rm T}_{j-1}}\right)\varepsilon , \widehat{{\rm T}_{0}}=0, \widehat{{\rm T}_{s+1}}=1\}$$

Is the set of permissible breaks in between the estimated $(j-1)$-th and j-th breaks. The above menthioned statistic is applied sequentially.

### Spectral causality

Finally, for robustness, we make use of spectral causality testing to assess the causal relantionhsips among the variables that enter the model in different volatility regimes. Spectral causality detects non-causal relatiohsips among variables based on changes in the frequency domain. See Konstantakis, et al., (2021), Breitung and Candelon (2006). The test can be used to determine whether a particular component of the “cause” variable at frequency ω is useful in predicting the component of the “effect” variable at the same frequency one period ahead.

Let $Y_{t} = \left( {x_{t} ,y_{t} } \right)^{^{\prime}}$, a covariance-stationary vector time series represented by a finite-order vector autoregressive model – VAR(p).

19 $$ \Theta \left( L \right)Y_{t} = \varepsilon_{t} $$

where $\Theta \left(L\right)={I}_{2}-{\Theta }_{1}L-{\Theta }_{2}{L}^{2}-\dots -{\Theta }_{p}{L}^{p}$ a lag polynomial with backshift operator${Y}_{i}{L}^{i}={Y}_{i-1}$, ${I}_{2}$ is the identity matrix;${\Theta }_{i}$, i = 1,2,…,p is a coefficient matrix associated with lag i and $\varepsilon_{t} = \left( {\varepsilon_{1t} ,\varepsilon_{2t} } \right)^{^{\prime}}$ denotes a vector white-noise process with $E\left({\varepsilon }_{t}\right)=0$ and positive-definite covariance matrix $\Sigma = E\left( {\varepsilon_{t} \varepsilon_{t}^{^{\prime}} } \right)$.By applying Cholesky factorization, $GG^{^{\prime}} = \Sigma^{ - 1}$, G being a lower-triangular matrix), we have a moving average representation of the system in Eq. (19):

20 $$ \left( {\begin{array}{*{20}c} {x_{t} } \\ {y_{t} } \\ \end{array} } \right) = \Phi \left( L \right)\varepsilon_{t} = \left( {\begin{array}{*{20}c} {\Phi_{11} \left( L \right)} & {\Phi_{12} \left( L \right)} \\ {\Phi_{21} \left( L \right)} & {\Phi_{22} \left( L \right)} \\ \end{array} } \right)\left( {\begin{array}{*{20}c} {\varepsilon_{1t} } \\ {\varepsilon_{2t} } \\ \end{array} } \right) = \Psi \left( L \right)\eta_{t} = \left( {\begin{array}{*{20}c} {\Psi_{11} \left( L \right)} & {\Psi_{12} \left( L \right)} \\ {\Psi_{21} \left( L \right)} & {\Psi_{22} \left( L \right)} \\ \end{array} } \right)\left( {\begin{array}{*{20}c} {\eta_{1t} } \\ {\eta_{2t} } \\ \end{array} } \right) $$

where, $${\eta }_{t}=G{\varepsilon }_{t}$$ $$E(\eta_{t} \eta_{t}^{^{\prime}} ) = I$$, $$\Phi \left(L\right)=\Theta {\left(L\right)}^{-1}$$ and$$\Psi \left(L\right)=\Phi {\left(L\right)G}^{-1}$$.

Applying Fourier transformation of the moving average polynomial terms, we rewrite the spectral density of ${x}_{t}$ as:

21 $$ f_{x} \left( \omega \right) = \frac{1}{2\pi }\left\{ {\left| {\Psi_{11} \left( {e^{ - i\omega } } \right)} \right|^{2} + \left| {\Psi_{12} \left( {e^{ - i\omega } } \right)} \right|^{2} } \right\} $$

Geweke’s measure of linear feedback from ${y}_{t}$ to ${x}_{t}$ at frequency $\omega$, is defined by:

22 $$ M_{y \to x} \left( \omega \right) = \log \left\{ {\frac{{2\pi f_{x} \left( \omega \right)}}{{\left| {\Psi_{11} \left( {e^{ - i\omega } } \right)} \right|^{2} }}} \right\} = {\text{log}}\{ 1 + \frac{{\left| {\Psi_{12} \left( {e^{ - i\omega } } \right)} \right|^{2} }}{{\left| {\Psi_{11} \left( {e^{ - i\omega } } \right)} \right|^{2} }} $$

If ${\left|{\Psi }_{12}\left({e}^{-i\omega }\right)\right|}^{2}=0$, then ${M}_{y\to x}\left(\omega \right)=0$. In this case ${y}_{t}$ does not Granger cause ${x}_{t}$ at frequency ω. The null hypothesis is the following:

$${H}_{0}:{M}_{y\to x}\left(\omega \right)=0$$

Breitung and Candelon (2006) showed that when ${\left|{\Psi }_{12}\left({e}^{-i\omega }\right)\right|}^{2}=0$, we also have ${M}_{y\to x}\left(\omega \right)=0$ and ${y}_{t}$ does not Granger cause ${x}_{t}$ at frequency ω if the following condition is satisfied:

23 $$ \left| {\Theta_{12} \left( {e^{ - i\omega } } \right)} \right| = \left| {\mathop \sum \limits_{k = 1}^{p} \theta_{12,k} cos\left( {k\omega } \right) - \mathop \sum \limits_{k = 1}^{p} \theta_{12,k} sin\left( {k\omega } \right)i} \right| = 0 $$

${\theta }_{12,k}$ is the (1,2)-element of ${\Theta }_{k}$. In this case, the necessary and sufficient conditions for $\left|{\Theta }_{12}\left({e}^{-i\omega }\right)\right|$ are: $\sum_{k=1}^{p}{\theta }_{12,k}cos\left(k\omega \right)=0$ & $\sum_{k=1}^{p}{\theta }_{12,k}\mathrm{sin}(k\omega )i=0$

Breitung and Candelon (2006) reformulated these restrictions by rewriting the equation for ${x}_{t}$ in the VAR(p) system:

24 $$ x_{t} = c_{1} + a_{1} x_{t - 1} + \ldots + a_{p} x_{t - p} + b_{1} y_{t - 1} + \ldots + b_{p} y_{t - p} + \varepsilon_{1t} $$

where ${a}_{j}={\theta }_{11,j}$ and ${b}_{j}={\theta }_{12,j}$. The null hyposthesis is equivalent to:

$${H}_{0}:R\left(\omega \right)b=0$$

where $b = (b_{1} , \ldots , b_{p} )^{^{\prime}}$ and $R\left(\omega \right)$ is a 2xp restriction matrix:

$$R\left(\omega \right)=\left[\begin{array}{cc}\mathrm{cos}\left(\omega \right)& cos(2\omega )\\ sin(\omega )& \mathrm{sin}\left(2\omega \right)\end{array}\begin{array}{cc} \dots & cos(2\omega )\\ \dots & \mathrm{sin}\left(2\omega \right)\end{array}\right]$$

Due to the fact that there are linear restrictions, the usual Wald statistic can be used. Let $\gamma = \left[ {c_{1} ,a_{1} , \ldots ,a_{p} ,b_{1,} \ldots ,b_{p} } \right]^{^{\prime}}$ be a $q=\left(2p+1\right)x1$ vector of parameters, and let V be a qXq covariance matrix from the unrestricted regression (24). The Wald statistic is the following:

25 $$ W = \left( {Q\gamma } \right)^{^{\prime}} \left( {QVQ^{\prime}} \right)^{ - 1} \left( {Q\gamma } \right)\sim X_{2}^{2} $$

where *Q* is a 2xq restriction matrix: $Q=\left[{0}_{2X\left(p+1\right)} \vdots R(\omega )\right]$

## Empirical analysis

### Data and variables

Our daily dataset covers the period from 1 January 2020 until 1 January 2021, fully capturing the recent Covid-19 pandemic. The prices of the S&P 500 stock index, the 10-year US benchmark government bond index, and the carbon dioxide emissions allowances (EUAs) are retrieved from Refinitiv Eikon Datastream. All price data have been transformed into daily returns, using the formula (see e.g. Michaelides, Tsionas and Konstantakis, 2016):

26 $$ Returns_{{p_{t} }} = \ln \left( {\frac{{P_{t} }}{{P_{t - 1} }}} \right), t = 1, \ldots T $$

The data on the Covid-19 new cases are also in daily frequency and come from the Johns Hopkins University database, which is freely accessible to the public.

Table 1 below provides a compact description of the data.

**Table 1:** Definition of variables

Variable	Description
Returns_SP500	The returns of S&P500 as calculated by the S&P500 price index, using the formula in equation twenty six (26)
Returns_Emissions	The returns of the the carbon dioxide emissions allowances (EUAs) as calculated by the formula in equation twenty six (26)
Returns_US_Bonds	The daily price of the 10-year bond yields for the US economy
Returns SP500 (top 10%)	The Returns_S&P500 variable where its observations lie at the top 10% quantile
Returns US Bonds (top 10%)	The Returns_US_Bonds variable where its observations lie at the top 10% quantile

### Date stamping of the two covid-19 waves

Throughout the entire ongoing period of the pandemic, the preferences of investors and firms have changed based on each wave of the pandemic, since different economic and lockdown measures have been implemented in each wave. Therefore, in order to capture these shifts, and extract vital information rgerading the expected future quantity of carbon emmisions, we need to extend our analysis by capturing the two waves in the Covid-19 era that span our dataset. In order to accurately time stamp the two waves, we make use of the popular state-of-the-art sup-ADF test by Phillips and Shi (2020) and Phillips et al., (2011, 2015).

**Fig. 1:**
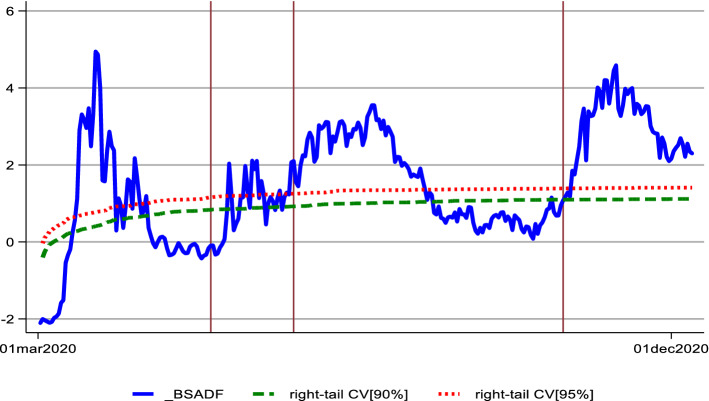
Date Stamping of the two Covid-19 Waves. The figure presents the results of the Bootstrapped Supremum ADF test for the new cases of Covid-19. The cut-off days marked, designate the two waves of the Covid-19 pandemic

Figure 1 presents our findings. Note that since we are interested in dating the two wave periods of Covid-19 and not the explosive behaviour of Covid-19, based on the sup-ADF test, we will also include in each wave the beginning and the end of the explosive nature of the Covid-19 pandemic. This dating choice will allow us to model and capture all the points that lie below the sup-ADF threshold, using the low volatility state of our Markov-Switching (MS) approach. Based on Fig. 1, the first wave begins on the 24th of January 2020 and ends on the 15th of May 2020, whereas the second wave begins on the 31st of July 2020 and ends on the 17th of November 2020.

The red markers in Fig. 1 indicate the cut-off dates for the two Covid-19 waves.

For robusteness, we make use of the Bai-Perron structural Break Test for known dates of the breaks in order to validate our findings. Table 2, presents the results of the test. Based on the structural break test results, the dating of the two waves of the Covid-19 pandemic are econometrically robust.

**Table 2:** Bai-Perron Structural break Test for known dates

Test statistic		Bai-Perron critical values		
		1% critical value	5% critical value	10% critical value
SupW (tau)	178.90	6.19	4.99	4.41

Estimated Breaking Points: 24/1/2020; 15/05/2020; 31/07/2020; 17/11/2020. Trimming: 0.10

Having determined the two waves of the pandemic, we present the variables’ descriptive statistics for each wave in Table 3. We observe remarkable differences between the two waves, with lower return volatilities across all three asset classes during the second wave. In fact, it is worth noticing that during the first wave, the average returns of futures of the carbon emissions are the highest among the alternative investments in the US stock market and/or the 10 year US bonds. This, in turn, gives us a first sign of an increase in the expected quantity of total emissions during the first wave of the pandemic. The statistical significance of these differences will be econometrically assessed by the Markov-Switching (MS) model employed.

**Table 3:** Descriptive Statistics for the two Covid-19 waves

Variables	Mean	Std. Dev	Min	Max	Skew	Kurt
*1st Covid-19 wave*						
Returns_Emissions	− 0.003	0.041	− 0.018	0.129	− 0.736	8.027
Returns_SP500	− 0.002	0.035	− .128	0.090	− 0.385	5.397
Returns_US_Bonds	0.001	0.008	− 0.024	0.021	− 0.596	5.812
*2nd Covid-19 wave*						
Returns_Emissions	0.001	0.029	− 0.066	0.075	− 0.735	8.028
Returns_SP500	0.001	0.012	− 0.036	0.022	0.385	5.396
Returns_US_Bonds	− 0.001	0.003	− 0.013	0.011	− 0.596	5.816

Emissions’, ‘SP500’ and ‘US_Bonds’ denote the three assets under investigation, that is the carbon dioxide emissions allowances (EUAs), the S&P 500 stock index and the 10-year US benchmark government bond index, respectively

Next, we proceed with the estimation of the MS model for the two waves of the pandemic. The results of our analysis, reported in Table 4, show that in the high volatility state in the first wave, which captures the increased market turmoil, carbon emissions do not exhibit a safe haven behavior. Nonetheless, carbon emissions seem to act a hedge against the stock market returns and against the US bonds, since the respective coefficients are negeative and statistically significant in the low volatility state.This, in turn, implies that investors expect that the quantity of carbon emissions will increase, i.e. a rebound effect in carbon emissions is expected by the market actors.

**Table 4:** Markov-Switching (MS) estimation results across the two Covid-19 waves

Independent variables	Returns on emissions			
	1st Wave		2nd wave	
	Low volatility state	High volatility state	Low volatility state	High volatility state
Returns SP500	0.628***	0.946***	1.777***	1.338***
	(15.28)	(7.25)	(5.56)	(4.22)
Returns US Bonds	3.375***	− 0.210	− 2.774***	1.989
	(27.79)	(− 0.41)	(− 6.37)	(0.85)
Returns SP500 (top 10%)	− 0.0545***	− 0.000973	− 0.0251***	− 0.0330**
	(− 11.06)	(− 0.07)	(− 4.50)	(− 2.39)
Returns US Bonds (top 10%)	− 0.0433***	0.00662	0.0129*	− 0.0192
	(− 9.26)	(0.65)	(1.72)	(− 1.03)
Returns SP500 (− 1)	1.793***	− 0.0823	− 0.360**	1.167**
	(10.21)	(− 0.61)	(− 2.54)	(3.02)
Returns US Bonds (− 1)	− 0.108***	− 0.745	0.199	2.066*
	(− 3.86)	(− 1.37)	(0.30)	(2.16)
Constant	− 0.0147***	0.00781*	− 0.0214***	0.0200***
	(− 9.58)	(2.18)	(− 7.80)	(4.26)
Ln volatility (σ)	− 5.568***	− 3.776***	− 3.993***	− 4.602***
	(− 14.47)	(− 31.17)	(− 43.95)	(− 42.09)

t-statistics in parentheses, *p < 0.10, **p < 0.01, ***p < 0.001

Top 10% implies the observations that belong to the lower 10% quantile; (-1) indicates the first lag of each variable

Turning to the second wave of the pandemic, the results show that carbon emissions seem to act as a safe haven against stocks in the high volatility state, since the respective coefficient is negative and statistically significant. For the low volatility state, the picture remains the same as in the first wave, since carbon emissions act as a hedge against both US stocks and US bonds. Note, that the positive and statistically significant coefficient of the US bonds is very close to zero, and thus, a hedge behavior is in force. Therefore, in a sustainability perspective, during the second wave, investors still expect that the quantity of carbon emissions will rise in the future.

The difference between the two waves could be attributed to various facts. In the first wave, the lockdown measures implemented, the travel restrictions, as well the characterization of Covid-19 as a global pandemic by the World Health Organization (WHO), spread fear among investors since the unfolding of the pandemic was unprecedented. In addition, in the first wave, the overall financial risk for all financial institutions and economies was very high since the rescue packages of ECB and Federal Reserve bank were finalized at the end of April. On the other hand, in the second wave, the policy responses were almost the same and even in some cases milder than those of the first wave, whereas the overall financial risk was relatively low compared to the first wave given that the rescue packages were already in place.

In order to empirically verify the behaviour of carbon emission returns during the two waves, we need to estimate the correlation between emission, bond, and stock index returns. In this context, Table 5 reports the correlation coefficients as well as their statistical significance. The behavior of carbon emissions as a hedge commodity in the first wave, and as a safe haven via a vis the US stock returns in the second wave is verified, according to the three asset behaviour types described earlier.

**Table 5:** Correlation coefficients

	1st Covid-19 wave			2nd Covid-19 wave		
	Returns emissions	Returns SP500	Returns US bonds	Returns emissions	Returns_SP500	Returns US bonds
Returns Emissions	1.000			1.000		
Returns SP500	0.177	1.000		0.216	1.000	
Returns US Bonds	− 0.129	− 0.149	1.000	− 0.179	0.014	1.000

The table presents the pairwise correlation coefficients between the variables between the two Covid-19 waves

Next, we estimate the expected duration of the two volatility regime states. Our findings in Table 6 demonstrate quite striking differences across the two waves of the pandemic. In the first wave, the high volatility state has an expected duration of approximately one and a half days, whereas in the second wave the expected duration is somewhat smaller. Moreover, the low volatility state in the first wave is approximately four and a half days, i.e. almost three days more than the high volatility state. On the contrary, in the second wave, the low volatility state is approximately one and a half days, i.e. slightly higher than the high volatility state. These differences in the expected duration between the two waves highlight the financial market adaptability to the Covid-19 pandemic. In other words, the financial markets learn how to operate under the stress induced by the pandemic.

**Table 6:** Duration of the high and Low Volatility states between the two Covid-19 waves

State	1st wave of Covid-19	2nd wave of Covid-19
High volatility	1.413***	1.261***
Low volatility	4.307***	1.505***

The table presents the expected duration of each volatility state in the two Covid-19 waves

*p *< 0.10, **p < 0.01, ***p < 0.001

Finally, Table 7 presents the transition probabilities between the high and low volatility states across the two waves of the pandemic. A striking finding is that in the first wave the expected probability for moving to a low volatility state is over 70% irrespectively of the prior volatility state. However, in the second wave, the expected probability for moving to the high volatility state is over 65%, when our prior volatility state is the low one. In other words, in the second wave, we witness a high expected probability for moving to a high volatility state when the low volatility state is realized, i.e. sudden jumps to the high volatility state.

**Table 7:** Transition probabilities between high and low volatility states in the two CoVid-19 waves

State	1st Covid-19 Wave		2nd Covid-19 Wave	
	High volatility	Low volatility	High volatility	Low volatility
High volatility	0.292***	0.708***	0.209***	0.791***
Low volatility	0.232***	0.768***	0.665***	0.335***

The Table presents the expected probabilities for the transition between high and low volatility states for the two waves of Covid-19 pandemic

*p *< 0.10, ** *p *< 0.01, *** *p *< 0.001

It may well be that these sudden jumps in volatility can be explained by the fact that investors in the second wave were more prepared for increased turmoil and sudden jumps in volatility as compared to investors in the first wave. In other words, compared to the first wave, where investors were entirely unprepared because they had no prior knowledge about these things, the second wave of investors were quite well prepared from the beginning.

### Robustness

In order to provide a cross validation for our findings regrading the Markov-Switching estimation results, we employ spectral causality testing between the returns on emmisions and the returns of S&P 500 and 10 year US bonds, respectively.

Based on the results presented in Table 6, we reject the null-hypothesis of non-causality for the returns of S&P 500 and 10-year US bonds on the returns of emmissions, a fact that is consistent with our primary finding that carbon emissions acted as a safe-haven for investors in the first wave of the ongoing pandemic.

Turning to the second wave of the pandemic, based on Table 8, we can infer that a causal relationship between S&P 500 returns and returns on emissions is in place, whereas there is no-evidence of causality between emissions and 10-year US bonds.

**Table 8:**
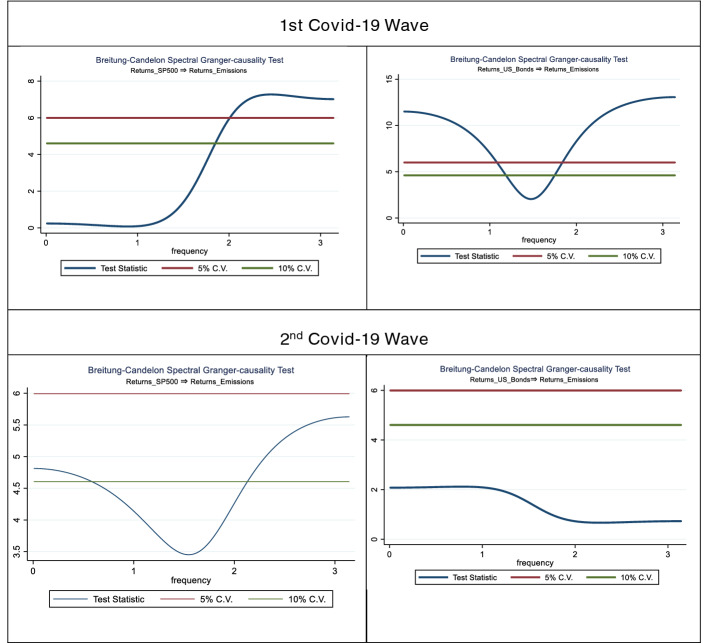
Spectral Non-causality tests of S&P500 and 10-year US bond returns on Emmission returs

In fact, Table 9, presents the spectral frequency of the spectral non-causality tests performed, as well as the duration of causality in days.

**Table 9:** Spectral range and Duration of causality

Causal variables	Range in rads (ω)		Range in time (days)	
1st wave Covid-19				
Returns_SP500	1.85	3.14	2	3.4
Returns_US_Bonds	0–1.18	1.75–3.14	3.59–3.14	5.32–80
2nd wave Covid-19				
Returns_S5P00	0–0.58	2.13–3.14	2.95–3.14	10.83–77

Based on Table 9, we observe that in the first wave the returns of S&P500 “cause” the evolution of the returns of emissions for 1.4 days. This causal relationship is also in force during the second wave of the pandemic, with the spectacular difference that its duration now lasts for more that 60 days. A fact that highlights that in the second wave of the pandemic, S&P500 dictates the evolution of the emissions for almost the entire second wave of the pandemic. However, for the returns of the 10 year US bonds we have almost the opposite picture, i.e. very long-lasting causal inference on the evolution of emission returns for the first wave and non-statistically significant inference for the second wave.

## Discussion and policy implications

Based on our findings, carbon emissions exhibit a hedge behaviour in both waves of the pandemic. This, in turn, implies that investors, comprised by firms and mutual funds, anticipate that the future returns of carbon emissions are expected to rise in the future. This rise in the total expected quantity of carbon emissions would be attributed to a wealth of factors.

To begin with, the lockdown measures implemented by the majority of policy actors across the world had a profound effect on various economic sectors, such as transportation, production and distribution. In the beginning of the pandemic, the aviation industry was heavly hit due to these measures, since the number of flights has been reduced globally by more than 40% because of the pandemic. This in turn, impacted the overall freight transportation by almost 20%, compared to 2019. As a result, the carbon emissions induced by freight transportation in general, declined in the pandemic era by by more than 20%. Turning to the production of industries, in a worldwide context, the overall reduction due to the pandemic and the confinement measures implemented was estimated to be roughly 35%. This, in turn, yielded a reduction of 19% in carbon emissions compared to 2019 (Quere et al. 2020).

Based on the aforementioned factors, it is quite natural to expect that in the post-pandemic era, the confinement measures will be alleviated, and this will lead transportation, production and distribution, at least back to their initial levels of economic activity. Therefore, from this point of view, it is natural to expect a rebound effect in the total quantity of carbon emissions. However, there are also a series of measures implemented by policy actors that could lead carbon emissions to levels that will be even higher in the post-pandemic era compared to 2019.

During the pandemic era, the US Enviromental Protection Agency (EPA) decrased substantially the standards of the average fuel efficiency in the car fleet of each automobile company from 5% to 1.5%. In addition, the EPA, in an attempt to boost production, it announced a relaxation of the environmental regulations and fines during the pandemic to industries that were affected by the pandemic. More precisely, the EPA removed the fines imposed to companies that failed to report, or meet the requirements for emitting pollutants. In fact, if a US industry was directly affected by the pandemic, then it could skip daily pollution inspections, tests and training (Wang & Li, 2021). Clearly, the policy actions undertaken from the US environmental policy makers, as a response to the pandemic give the incentive to industries to increase their overall in pollutants and substantially delay the decarbonization of the US economy. Nonetheless, unfortunately, US was not the only economy that took hazardous policy actions in terms of carbon emmisions, since the UK, as well as the EU announced various relaxations on the energy efficiency standards as well as on regulations regarding the operation of fossil based industries.

All the aforementioned evidence provide a clear indication of a strong rebound effect of carbon emissions in the post pandemic era. Therefore, the prevailing question, in a policy perspective, is how this rebound effect could be minimized or even avoided. Clearly, the answer to this important question is based on a variety of strict policy actions that need to be implemented. More precisely, as a first step, it is important, that policy actors across the globe should acknowledge the fact that the pandemic offered us with aν opportunity to exogenously (unplanned) reduce the overall amount of carbon emissions to a level copmarable to 2006 (Quere et al. 2020). Based on the related literature, carbon efficiency and resource efficiency are interchangeably linked (Trinks et al., 2020). As a result, policy makers should focus on tailor-made policy actions that would offer firms the incentive to become more resource-efficient, a fact that could be achieved with an increased level of circularization of industries. This circularization, in turn, will make firms more efficient in terms of resourses and thus more efficient in terms of their carbon emissions.

Additionally, it is important that policy makers acknowledge the important role of households in the reduction of carbon emissions (Li et al., 2019). The confinement measures of the pandemic and the adverse economic consequences led the majority of households to a more frugal lifestyle, characterized by decreased expenses in consumption and of course transportation. As a result, tailored made policy actions that would offer the incentive to households to maintain their level of consumption as well as incetives for using green transportation, such as bicycles and electric vehicles would have a direct beneficial impact on the level of carbon emissions.

Another step towards a rebound effect for the carbon emissions in the post pandemic era would be the supervised regulation of Emissions Trading System globally. Thus far, the EU regulatory framework on (ETS) despite its drawbacks, is quite efficient in terms of promoting low-carbon technological change in various industries (Teixido et al. 2019). In this context, policy makers should consider using the best practices from the EU ETS regulatory framework to heavily regulate ETS on a global scale.

## Conclusions

In the course of the epidemic, the global quantity of carbon emissions has decreased by 6.4%, reaching levels that are directly comparable to the levels reached in 2006 at the beginning of the epidemic. Nevertheless, the most important question in the face of this decline is whether this reduction could be maintained in post-pandemic times. Using the safe-haven methodology used in finance and adapting it to the context of environmental sustainability, we were able to extract information regarding investors’ beliefs regarding the amount of carbon emissions that will occur in the future in order to assess our research question.

Based on our analysis and the robustness checks that were performed on the future returns of carbon, it appears that emissions acted as a hedge with respect to both the performance of the US stock market and the performance of its bonds during both waves of the pandemic. In general, we believe that in a global context it is very likely that there will be a strong rebound effect for carbon emissions that occur as a result of what we have seen in our analysis. It is for this reason that this paper discusses the reasons behind this rebound effect in terms of policy interventions implemented and also suggests specific policy recommendations that could help minimize this effect in the future.

Of course, the decarbonization of economies in a global scale can only be achieved through collaboration and not by free-riding. Therefore, all the aforementioned policy actions suggested require a close collaboration of policy actors with the respective general goverments in each economy as well as with the representatives of carbon inefficient industries.

In a similar context, a great idea for future and more extended research would be to incoropoarate cryptocurrency assets in the model as well as to test whether a cryptocurrency asset, such as the bitcoin (BTC), could act as a safe-haven in the post pandemic era. This is an interesting subject that would be of special interest for future research and further study.
